# Synopsis of an Introductory Lecture to a Course of Dental Mechanics

**Published:** 1880-11

**Authors:** J. Walker

**Affiliations:** Dental Hospital of London


### SYNOPSIS OF AN INTRODUCTORY LECTURE TO A COURSE ON DENTAL MECHANICS.

BY DR. J. WALKER, IN THE DENTAL HOSPITAL OF LONDON, SESSION 1880 AND 1881.

To come to the special subject of my lecture. At the risk of provoking the well known retort of "nothing like leather," I venture to assert that no man can ever prove himself a good Dental Surgeon unless he is a skilled artist in Dental Mechanics. To kindle a spark of my own enthusiasm for the subject, to fan that spark into a flame that shall burn brighter and brighter in your life until you lay down the file and the engraver, with a sense that you have done some good work in the world, is at once my endeavor and my duty. A painter or sculptor of eminence in his profession is frequently the leader in the fashionable world, he is feasted by the city guilds, his company is sought by the rich and noble, he has the *entree* to the literary circles of every capital, yet the work of the artist is, at best, but to reproduce a faint imitation of nature in cold marble or on inanimate canvas. The subject of your handiwork will be full of life and animation.

What is the necessary training for such accomplishments? A full and perfect realization of all the forms of human beauty, and of woman's beauty in particular. Why do I dwell so much on the complete form of beauty? Because no face can be perfect in beauty, unless its features each and all are in harmony. The teeth have a peculiarly marked position in relation to the features: one missing link in the circle will attract attention and mar the harmony of an otherwise lovely face, like a false chord in music.

Projecting, discolored, irregular, misshapen, crowded teeth, all tend to destroy symmetry. If so in the natural course of dentition, how much more in the artificial!

I would urge you all to undertake at starting a thorough study of the normal bones of the skull.

Normal bones of the face.—In the anatomical class at your general hospital you will study the bones of the face in considerable detail, but there your attention will be directed to the common or general characters of the bones. You will there have to learn the usual shape of the bones, their processes, ridges, grooves and depressions; you will be shewn the characters, not only by which you may at once recognize them, but which you may always recognize in them. I cannot too strongly urge you to master all these details.

But, Gentlemen, here we have to study these bones in a practical manner; we have to look upon them as parts of the living countenances of our patients, and as no two faces are exactly alike, a study of individual faces is necessary, as a groundwork for your success in practical mechanical dentistry, and you must study individual specimens of each of the facial bones. A careless observer of a crowd of negroes might think they were all alike, because each had a black skin, woolly hair, retreating foreheads, thick lips and white teeth. But yet a close observation would quickly tell him in truth there came behind all these coarse resemblances, minute, but noteworthy differences, differences which he would be compelled to take note of before intercourse with them would be possible. In the same way a general anatomist merely points out to you how all palates are alike, and I want to go farther and shew you with equal truth how no two are alike, but all differ. Depend upon it, gentlemen, your usefulness and successes will vary with your skill in perceiving these lesser differences which characterise individuals. The best name I can give to this study is comparative human anatomy.

Let us take some examples of what I mean. The upper jaw bone is the most complex of all the bones of the face. Looking at its central part or body we are first of all struck with the cavity in it—the antrum of Highmore. How, if you take a hundred bones, you will not be able to find two antra exactly alike, but they will differ in size, in shape, in depth, in width, and in size of their angles and inclination of each of their walls. And all these peculiarities influence the countenance, and must, therefore, be studied before you can hope to be successful in replacing the lost dental organs. The high cheek bone of the Scotchman is a very familiar example of the effect of a variation in the antrum.

The alveolar process.—The natural setting of the teeth varies also in its depth, thickness, smoothness, irregularity, and most improtantly in its curve, which may be a broad, open semi-circle, or a narrow semi-ellipse. The nasal process, too, varies as much in different specimens; you will find differences in length, breadth, in the angle it forms with the body of the bone, and with the frontal bones; all these particulars modify the shape of the nose, and as I shall have to point out to you, no feature is more worthy of your careful study than the human nose in its numberless varieties. The malar process of this bone has similar varieties. Notice again the palate plate how it differs in breadth and arch, and so modifies importantly the roof of the mouth, to which a denture has been adapted. The malar bones are unlike in thickness, the size of their angles, length of their offspringing processes, and in the exact mode of articulation with neighboring bones. See too, how frontal bones vary, in one case a broad, bold line forehead, in another overhanging, in a third narrow and pointed, and you meet with infinite varieties between these extremes.

In passing to the nasal bones, not only must we notice how they differ in length and breath, and the level of their edges, but that the shape of their arch is constantly varying; it may be broad and rounded, or narrow and high, even to sharpness. This depends upon the prominence forward of the bony nasal septum, the interval between the nasal processes of the upper maxilla, that is to be bridged over the breadth of the nasal bones, and the exact mode of their articulation with the upper jaw bone. Not alone does the usual arch differ thus, but most obviously on the angle it forms with the frontal lines.

From your own observation you will at once grant me that noses vary as much as families; in fact I am inclined to think that there is a good deal to be said for Mr. Shandy's philosophy of noses. The cartilages of the nose play a most important part in tbe shape of the organ, and demand your study as much as the bones. Each variation in the shape of the nose has a corresponding variety of upper lip, and the correlations between these two must be most carefully attended to. Granted that these differences are so numerous, you must admit that the nose must have primary importance in the estimate of the Dental Surgeon, when called upon to restore the lost Dental organs. I may remark that although I am examining noses every day of my life, I have never yet found one assuming a direct line with the other central lines of the head and face.

To arrive at a just appreciation of the effect of these bones on the lines of the face you must examine them in the articulated skeleton, not in one instance but in many—fifty or even a hundred—make weekly visits to the Museum of the Royal College of Surgeons, and there examine all the specimens of articulated skulls and skeletons, until you fully grasp the meaning of comparative human anatomy—the size, the shape, the relative acuteness of angles, the proportions of the different parts. It is this relationship, the articulation of each bone with the other bones of the skull that is of primary importance to the Dental Surgeon.

The last bone that I shall mention to you this evening is the lower jaw, perhaps the most important of all. You will all soon be taught that it has a body, a symphisis, a ramus with its condyle, coronoid process and sigmoid notch, and alveolar process, and various tubercules, ridges, spines, grooves, and depressions. But beyond all such facts, be at pains to notice, gentlemen, how all these various parts differ in different specimens. The changes in the angle of the bone that are met with at different ages are notorious, but you will have to learn that the angle of every adult differs, that each form of countenance has its special maxillary angle, nor are the depth, thickness, curve, obliquity and relative prominence of different parts of the bone one whit more constant, and if you would succeed in fitting artificial dentures to a lower jaw, these individual peculiarities of the bone must be carefully studied.

The Dental organs will be presented in full detail by my colleague, Mr. C. S. Tomes, but I should fail in the one point of my brief sketch, if I did not refer you to the fact that no circle is found exactly corresponding with a second in the articulation of the thirty two teeth implanted in the maxillary bones. My remarks culminate in this apparently strange contradiction, no two sets of teeth ever describe the same circle at any age. The differences in children are only slight, yet a difference exists; the older the subject, the greater the contrast visible. Yet harmony exists in the lines of the face; once acknowledge this and you will perceive the labor and investigation necessary to make you grasp the subject in its broadest sense.

Take, for instance, a patient at the age of sixty, with endentulous jaws, requesting artificial dentures to be prepared at your hands. For such mechanism to be successful in the restoration of the contour of the face, it will be necessary that it shall harmonize with the features; you must carry your perception backwards to the appearance that this face presented when he had only attained the age of thirty: this will be the art and science expected at your hands.

Not to lengthen this my introductory lecture, by laboring to define the various types of English faces, when the bones are covered with the soft parts, I have selected a few outlines of faces such as a painter and sculptor would study. Cast your eye upward, you will see that each possesses its own characteristic and alterable features. So in life; and if you gentlemen are to be true Dental Surgeons, you must rise to the ideal of artistic mechanics. If I am to benefit you to the full bent of my wishes, you must study nature in all its variety—nature when presented to you as destroyed by premature disease and death to be restored by your hand to its original conformation. Remember that use must follow beauty, one cannot be dissevered from the other if you would obtain results, complete in power of mastication, speech, durability, and appearance.

To win success in appearance, you must study the irregularities of the natural projection of circle, and the character of circle. If any irregularities are observed, take a model cast of the mouth, so that you can compare the natural organs whilst the artificial are in progress of arrangement; modify these irregularities, but do not efface their existence altogether. The general conformation of the Dental organs and the face must be your special study. The color of the teeth to be selected must receive at your hands great care: compare the various tints at your disposal, ascertain if a perfect self color, or a tinted, shaded, or stained tooth is the most pleasing; which will harmonize best with the complexion, producing a natural effect. It will be your object to disguise that the new introductions are foreign bodies, endeavor to make them appear as if they possessed life. Many colors absorb so much light that at night the appearance they present is black and death like. Avoid such shade, select those that reflect light; in many cases the teeth should be almost transparent. The color of the hair, the nature of the complexion will guide you in this endeavor; hair and complexion must harmonize. A heavy, waxy head of hair, dark and massive, with bronzed face would indicate a strong shaded tooth, solid in character and non transparent, yet a color that will reflect rather than absorb the rays of light. A Saxon face with fair hair will strongly puzzle you at times; the pearl hued, thin and transparent teeth, as a rule, are appropriate for such a face.

When articulating the dentures, every care must be taken and much thought and study bestowed to adjust the depth and height of the superior and inferior dentures to the length and depth, to the thickness or thinness of the lips. Note and estimate the loss of structure by absorption, and supply in proportion to the loss.

The circle and projection as above alluded to will be your special study. Give hours to produce a natural expression. Propose to the patient a short walk in your operating room; if a good corridor is at your disposal, so much the better, use it. By so doing you will ascertain what, if any, old habit of contortion of features is indulged in by the patient; the character of the laugh, if the lips are raised, and how much; at times only the tips of the teeth are manifest, at others the full lip is raised exposing to view the crown of the tooth and alveolus, even to the lower margin of the meatus of the nose. Arrangements equal to all these expressions must be adopted. The how, will be told in your future lectures.

Then, again, the smile, the smile of a Desdemona and the smile of an Iago! yet how much in a smile! Harmonize your mechanism so that the smile of the patient shall be rendered as natural and full of meaning as art can make it. The movements of the lips and tongue in speech must be carefully studied ; the eloquence of the orator and the no less eloquent prattle of a pretty woman. During the visit of your patient strike out a conversation that shall put him at his ease, and show him at his best; this will aid you in observing what is required to render conversation easy to himself and acceptable to his hearers. Notice that the head is never carried in a perpendicular line with the body; at times it leans to the right or the left, the effect of this inclination is to lengthen in appearance the teeth of the incline. Notice also, many patients have the ungainly habit of twisting the lips out of the natural line of the head and face.

It is by attention to all these details that the results of your work will be noble, your claims to reward great, your satisfaction not less in its kind than that of the sculptor or the painter; whilst they can only charm the happy, it is yours to relieve the suffering, to alleviate pain, and even to prolong life and restore lost beauty.

